# Efficacy of domperidone plus renal diet in slowing the progression of chronic kidney disease in dogs with leishmaniosis

**DOI:** 10.1186/s13071-022-05537-8

**Published:** 2022-10-31

**Authors:** Maria Alfonsa Cavalera, Floriana Gernone, Annamaria Uva, Rossella Donghia, Claudia Zizzadoro, Andrea Zatelli

**Affiliations:** 1 Department of Veterinary Medicine, University of Bari, Valenzano, Italy; 2 Unit of Research Methodology and Data Sciences for Population Health, “Salus in Apulia Study” National Institute of Gastroenterology “S. de Bellis” Research Hospital, Bari, Italy

**Keywords:** Canine, Chronic kidney disease, *Leishmania infantum*, sSDMA, Serum creatinine

## Abstract

**Background:**

Chronic kidney disease (CKD) represents the main cause of mortality in dogs with leishmaniosis. Domperidone has recently been reported to improve kidney function in leishmaniotic dogs affected by CKD. Serum symmetric dimethylarginine (sSDMA) has also been shown to be a useful biomarker for earlier detection of decreased kidney function when compared to serum creatinine (sCr). This study aimed to assess the efficacy of domperidone plus renal diet in slowing the progression of nephropathy in leishmaniotic dogs with CKD, evaluating sSDMA and sCr as markers of kidney function.

**Methods:**

This study was a therapeutic, prospective, randomized, controlled, 11-month-long field trial. Dogs were recruited if classified as “exposed” to or “infected” with *Leishmania infantum* and affected by CKD at early stages. After enrolment (T0), dogs were randomized into groups T (treatment) and C (control). All dogs were fed a renal diet and then followed up at 90 (T1), 210 (T2), and 330 (T3) days after inclusion in the study. At T1 and T2, dogs in group T received an oral suspension of domperidone (1 ml/10 kg once a day for up to 28 days).

**Results:**

Twenty-two dogs (i.e., *n* = 12 in group T and *n* = 10 in group C) completed the study. At T0, the entire population of enrolled dogs presented a mean sSDMA value of 16.5 ± 3.4 μg/dl. At T1 (i.e., after 3 months of renal diet), sSDMA was significantly decreased in both groups, with an sSDMA of 13.1 ± 4.4 μg/dl for the entire population involved. From T1 to T3, sSDMA gradually increased in group C, while remaining stable in group T, which continued to show a significantly lower value of sSDMA at T3 than at T0. Regarding sCr, at T0 and T1, the mean values of the entire population of dogs were 1.1 ± 0.3 and 1.0 ± 0.4 mg/dl, respectively, with no statistical differences between groups T and C. In group T, sCr decreased significantly from T0 to T1, while returning at T3 to values similar to T0.

**Conclusions:**

In this study, domperidone plus renal diet reduced the progression of kidney disease in leishmaniotic dogs affected by CKD.

**Graphical Abstract:**

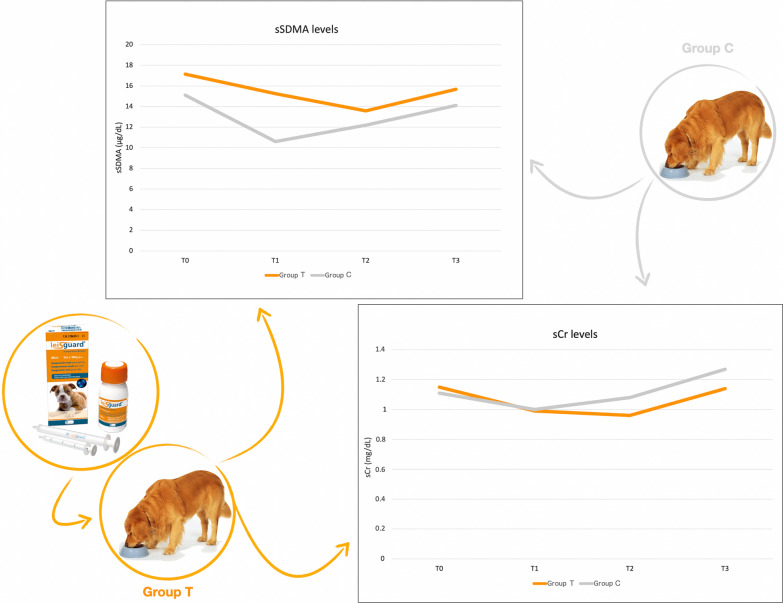

## Background

Canine leishmaniosis (CanL) is a sand fly-borne disease caused by infection with *Leishmania infantum*, whose diagnosis and treatment are still challenging for veterinary practitioners. Indeed, though the available protocols recommended by the main guidelines (i.e., LeishVet and Canine Leishmaniasis Working Group [CLWG]) [1, 2] can promote clinical cure and reduce the infectiousness of vector sand flies to dogs, the parasitological cure is rarely achieved, and the rate of clinical relapse is high [3–6]. Recently, immunostimulatory therapy has shown encouraging results against CanL, being able to improve dog cellular immune response and promote parasite reduction, while ameliorating clinical signs [7–9]. For example, the immunostimulatory drug domperidone, used as part of an integrated control program, is able to effectively manage the early stages of CanL or prevent the development of clinical disease, as it enhances innate/cell-mediated responses, potentiating the phagocytic and oxidative functions of canine neutrophils [7]. Furthermore, a recent therapeutic, prospective, and uncontrolled 7-month-long field study demonstrated that domperidone could maintain stable serum creatinine (sCr) and reduce anti-*L. infantum* antibody titres, globulins, gamma globulins, and C-reactive protein (CRP) in leishmaniotic dogs affected by chronic kidney disease (CKD) [10]. Therefore, the possible existence of two effects was hypothesized: (i) either a “direct” renal effect of domperidone, due to its specific dopamine 2 (DA_2_) receptor antagonist activity able to increase the glomerular filtration rate (GFR), renal plasma flow (RPF), and filtration fraction, potentially promoting renal filtration and maintenance of adequate sCr levels, or (ii) an “indirect” renal effect due to the improvement of CanL-related parameters [10].

During the last decade, serum symmetric dimethylarginine (sSDMA) has shown a good correlation with GFR and has been demonstrated to be a useful marker for earlier detection of decreased kidney function when compared to sCr [11–13]. Moreover, sSDMA seems a promising prognostic and predictive factor as a biomarker of kidney disease. Dogs with sSDMA of 15–19 μg/dl have a 2.5–4-fold higher risk of reduced renal function within 6 months than dogs with sSDMA ≤ 14 μg/dl [14].

In this scenario, this study aimed to assess the efficacy of domperidone plus renal diet in slowing the progression of nephropathy in dogs affected by leishmaniosis and CKD, evaluating sSDMA, sCr, and the ratio of sSDMA to sCr (sSDMA/sCR) as markers of kidney function.

## Methods

This study was a therapeutic, prospective (from October 2020 to August 2021), randomized, controlled, 11-month-long field trial conducted in a rescue shelter in the province of Lecce (40.419326°N, 18.165582°E; Apulian region, southern Italy), where CanL is endemic [15]. Dogs of any sex, age, weight, and breed were recruited for participation in this study if *L. infantum*-seropositive (i.e., antibody titre ≥ 1:80) by indirect fluorescent antibody test (IFAT), if classified as “exposed” to (i.e., stage A) or “infected” with (i.e., stage B) *L. infantum* according to the CLWG staging system [16], and if affected by early stages of CKD according to the International Renal Interest Society (IRIS) guidelines [17]. In this regard, CKD staging was based on fasting blood creatinine and fasting blood SDMA, and dogs classified as IRIS CKD stage 1 (i.e., sCr < 1.4 mg/dl) with sSDMA > 18 μg/dl and as IRIS CKD stage 2 were included [17]. Dogs were excluded if suspected of being or known to be affected by (i) co-infections with other vector-borne pathogens, such as *Ehrlichia canis* and *Anaplasma phagocytophilum*; (ii) diseases able to determine the progression of CKD (e.g., neoplastic, autoimmune, and heart diseases, diabetes mellitus or insipidus, hypo- and hyperadrenocorticism, or hyper- and hypothyroidism); (iii) chronic renal insufficiency of pre-renal or post-renal origin, nephropathy of toxic origin (over the previous 28 days); (iv) clinical signs compatible with lower urinary tract diseases; or (v) active forms of CanL (i.e., stages C–D according to the CLWG staging system) [16]. The same criteria were applied to exclude patients during the execution of the study. All dogs that had been administered either leishmanicidal or leishmaniostatic treatments in the previous 6 months were also excluded.

At inclusion time (T0), each dog was physically examined and a clinical sign-based score for CanL ranging from 0 to 19 was assigned (Table 1) [18]. Blood samples were collected from either the cephalic or jugular vein and placed in a tripotassium (K3) ethylenediaminetetraacetic acid (EDTA) tube (2 ml) to undergo routine haematology, and in a plain tube (5 ml) to obtain serum after centrifugation (15 min at 1500×*g*). For each enrolled dog, a complete blood count (CBC) with reticulocyte count, and a complete biochemical panel including acute-phase proteins (i.e., CRP), serum capillary electrophoresis, and sSDMA concentration measurement were performed.

**Table 1:** Clinical sign-based score for canine leishmaniosis ranging between 0 (i.e., absence of clinical signs) and 19 (modified from [18])

Systemic signs	Behaviour	Active	0
		Apathetic	1
	Body condition score (BCS)	3–5/5	0
		2/5	1
		1/5	2
	Muscle condition score (MCS)	1/4	0
		2/4	1
		3/4	2
		4/4	3
	Lymph nodes	Normal	0
		Enlarged	1
	Mucous colour	Normal	0
		Pale	1
	Bleeding (e.g., epistaxis)	Absence	0
		Presence	1
Cutaneous signs	Coat	Shiny	0
		Opaque	1
	Alopecia	Absence	0
		Presence	1
	Skin lesions	Absence	0
		Presence	1
		Ulcer	2
	Muzzle depigmentation	Absence	0
		Presence	1
	Muzzle/ear lesions	Absence	0
		Presence	1
	Nails	Normal	0
		Onychogryphosis	1
Ocular signs	Blepharitis	Absence	0
		Presence	1
	Keratoconjunctivitis	Absence	0
		Serous	1
		Mucopurulent	2

**Fig. 1:**
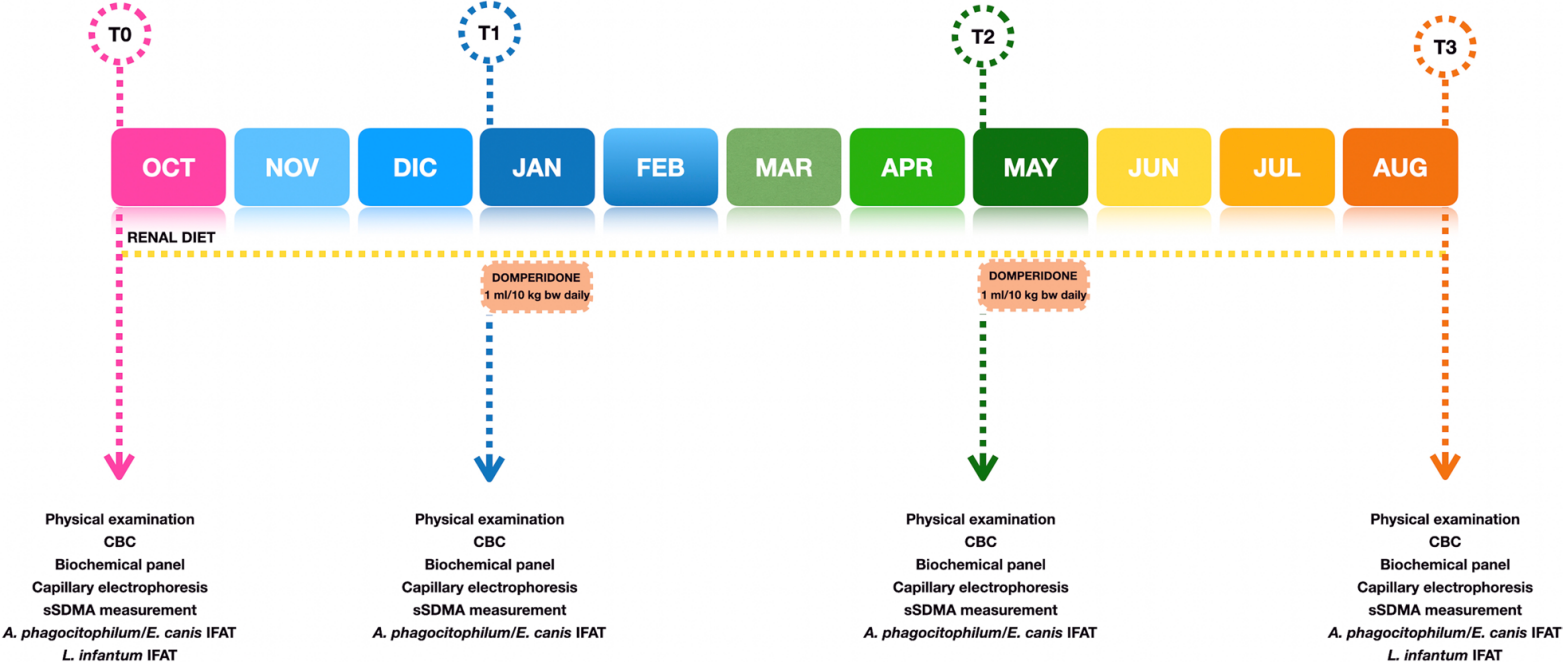
Timeline of the study project including time points, scheduled procedures, and treatment protocol. Abbreviations: CBC, complete blood count; IFAT, indirect fluorescent antibody test; sSDMA, serum symmetric dimethylarginine

Following inclusion, dogs were randomized into groups T (treatment) and C (control) and fed a renal diet which was administered according to the manufacturer’s instructions throughout the clinical study. Then, dogs enrolled were followed up at 90 (T1), 210 (T2), and 330 (T3) days after inclusion in the study, as shown in Fig. 1.

Dogs in group T received an orally administered suspension of domperidone (leisguard^®^: Ecuphar Italia srl, Milan, Italy) according to the following dosage and scheme: 1 ml/10 kg (0.5 mg/kg) orally once a day for up to 28 days starting at T1, 3 months without treatment, and 1 ml/10 kg (0.5 mg/kg) orally once a day for up to 28 days starting at T2 (Fig. 1). Product intake and the appearance of any side effects [19] were checked throughout the administration period by the kennel veterinary practitioner.

Furthermore, at each time point, dogs were tested for anti-*A. phagocytophilum* (MEGACOR Diagnostik, Horbranz, Austria) and anti-*E. canis* (Biopronix Agrolabo, Scarmagno, Italy) antibodies by IFAT. Finally, animals were checked for anti-*L. infantum* antibodies by IFAT at T0 and T3 [20].

## Statistical analyses

The normal distribution of the results was checked by the Kolmogorov–Smirnov test. sSDMA, sCr, and sSDMA/sCr values were normally distributed and reported as mean ± standard deviation (M ± SD), and as frequencies and percentages (%) for sSDMA categories. Linear mixed models with repeated-measures analysis were used to compare groups over time for each of the outcome measures. We modelled the random effects of dogs and fixed effects of group for baseline differences between groups. Comparisons were made for the dependent variables between treated and untreated groups, and with between-subject factor of time (baseline, T1, T2, and T3) as the repeated factor. In the case of interactions, the effects of the group at repeated factors of time were compared. For testing the associations between events at time point and SDMA categories, Fisher’s exact test for categorical variables was used, while to test equality for matched data, the McNemar–Bowker test was used. Furthermore, for testing the differences in sSDMA values between ordinal time groups for repeated measures in both groups T and C, the Friedman test was used.

When testing the null hypothesis of no association, the probability level of error at two tails was 0.05. All the statistical computations were performed using Stata statistical software (Release 17; StataCorp LLC., College Station, TX, USA).

## Results

Thirty mixed-breed dogs were initially enrolled in the study. Eleven dogs were neutered males and 19 spayed females, aged from 3 to 13 years, and weighing from 12 to 38 kg. At T0, animals were randomly assigned to either the T (treatment; *n* = 15) or C (control; *n* = 15) group by a blinded operator using Microsoft Excel. During the study period, several dogs were lost to follow-up due to causes either related or unrelated to CanL:

- In group T, two dogs were excluded because of recurrence of active CanL (i.e., at T3), and one dog died (i.e., at T3).
- In group C, one dog died of renal causes (i.e., at T2) and four dogs were excluded because of adoption and loss to follow-up due to non-compliance of the new private owners (i.e., *n* = 1 at T1 and *n* = 3 at T3).

The population completing the clinical trial was composed of 22 dogs (i.e., *n* = 12 in group T and *n* = 10 in group C). The effect of domperidone at the different time points on sSDMA, sCr, and sSDMA/sCR in group T compared with group C is shown in Table 2. The number of dogs grouped according to sSDMA (≤ 14, 15–19, ≥ 20 μg/dl) [14] at T1, T2, and T3 is shown in Table 3.

**Table 2:** Linear mixed model analysis to examine the effect of domperidone on serum symmetric dimethylarginine (sSDMA), serum creatinine (sCr), and sSDMA-to-sCr ratio (sSDMA/sCr) in group T (treatment) compared with group C (control) at different time points (i.e., T0–T3)

Parameters^*^	Time points				*P*^§^		
	T0 (a)	T1 (b)	T2 (c)	T3 (d)	(b) vs (a)	(c) vs (a)	(d) vs (a)
sSDMA (μg/dl)							
Group T	17.15 ± 2.77	15.25 ± 3.65	13.58 ± 3.37	15.67 ± 2.27	0.01	< 0.001	0.03
Group C	15.10 ± 3.60	10.60 ± 3.89	12.20 ± 4.66	14.10 ± 4.84	< 0.001	0.02	0.59
*P*^†^	0.05	0.003	0.44	0.37			
Mixed^‡^	Treatment 0.04	Time < 0.0001	Interaction 0.07				
sCr (mg/dl)							
Group T	1.15 ± 0.17	0.99 ± 0.16	0.96 ± 0.16	1.14 ± 0.20	0.02	0.01	0.90
Group C	1.11 ± 0.36	1.00 ± 0.64	1.08 ± 0.75	1.27 ± 0.94	0.27	0.90	0.03
*P*^†^	0.42	0.75	0.71	0.68			
Mixed^‡^	Treatment 0.93	Time 0.0002	Interaction 0.24				
sSDMA/sCr							
Group T	15.78 ± 3.93	15.39 ± 2.95	14.29 ± 3.42	13.96 ± 2.54	0.69	0.13	0.06
Group C	13.88 ± 2.01	11.80 ± 4.02	12.58 ± 4.31	12.64 ± 3.22	0.13	0.42	0.45
*P*^†^	0.15	0.02	0.35	0.51			
Mixed^‡^	Treatment 0.08	Time 0.26	Interaction 0.42				

^*^As mean and standard deviation (M ± SD)

^†^Treatment effect for each time

^‡^Mixed effects

^§^Contrasts of marginal linear predictions

**Table 3:** The number of dogs grouped according to serum symmetric dimethylarginine (sSDMA) (≤ 14, 15–19, ≥ 20 μg/dl) [14] at T1, T2, and T3

Parameters ^*^		Time points						Comparison^‡^	
		T1	*P*^†^	T2	*P*^†^	T3	*P*^†^	T2 _vs_ T1	T3 _vs_ T1
sSDMA			0.03		0.99		0.48		
Group T	≤ 14 μg/dl	5 (41.67)		9 (75.00)		5 (41.67)		0.17	0.26
	15–19 μg/dl	5 (41.67)		1 (8.33)		6 (50.00)			
	≥ 20 μg/dl	2 (16.67)		2 (16.67)		1 (8.33)			
Group C	≤ 14 μg/dl	9 (90.00)		8 (80.00)		7 (70.00)		0.32	0.16
	15–19 μg/dl	0 (0.00)		1 (10.00)		2 (20.00)			
	≥ 20 μg/dl	1 (10.00)		1 (10.00)		1 (10.00)			

^*^ As frequency and percentage (%)

^†^Fisher’s test

^‡^ McNemar–Bowker test

At enrolment (T0), the entire population of dogs involved in the study presented a mean sSDMA value of 16.5 ± 3.4 μg/dl, with group T having a higher mean value than group C (Table 2). At T1 (i.e., after 3 months of renal diet), sSDMA was found to be significantly decreased in both groups T and C (Table 2), with a mean sSDMA value of 13.1 ± 4.4 μg/dl calculated for the entire population of dogs involved in the study. At any rate, at this time point (i.e., T1), the sSDMA value of group T was significantly higher than that of group C (Table 2), and this was accompanied by a significantly higher sSDMA/sCr ratio in group T compared with group C (*P* = 0.02; Table 2).

From T1 to T3, sSDMA gradually and significantly increased in group C (Friedman Chi-square, *χ*^2^ = 10.757, *df* = 2, *P* = 0.004615), while remaining stable in group T (Friedman Chi-square, *χ*^2^ = 4.8261, *df* = 2, *P* = 0.08954) (Table 2). Accordingly, at T3 there was no statistical difference in sSDMA (*P* = 0.37) or sSDMA/sCr (*P* = 0.51) between the two groups, and sSDMA continued to be significantly lower than T0 in group T (Table 2).

Regarding sCr, at T0 and T1, the mean values of the entire population of dogs involved in the study were 1.1 ± 0.3 and 1.0 ± 0.4 (mg/dl), respectively, with no statistical differences between groups T and C (Table 2). In group C, sCr remained stable from T0 to T1, and then progressively increased, being significantly higher at T3 compared to T0 (Table 2). In group T, sCr decreased significantly from T0 to T1, and this decrease remained stable up to T2; then, by T3, the sCr had returned to a value similar to that recorded at T0 (Table 2).

Considering the cut-off of a 15% variation in sCr value as the limit for assessing a stable renal function in dogs [21], at the end of the study 8/10 (80%) and 6/12 (50%) dogs in groups C and T, respectively, showed a greater than 15% increase in sCr compared to T1.

sSDMA/sCr, although not showing a statistical difference, was lower at T1, T2, and T3 than at T0 in group T (Table 2).

During the trial, the dogs in both groups maintained a good state of health (i.e., clinical score = 0), except for one dog which presented a slightly higher clinical score of 2 (at T0 and T1) and 3 (at T2 and T3).

All dogs that completed the study tested negative for anti-*A. phagocytophilum* and anti-*E. canis* antibodies by IFAT at each time point. No side effects related to domperidone administration were observed.

## Discussion

In the past, the intrarenal administration of specific DA_2_-receptor antagonists proved effective in enhancing GFR, RPF, and filtration fraction in dogs [22]. Furthermore, a recent pilot study showed that oral administration of domperidone, a peripheral DA_2_-receptor antagonist, can improve renal function, antibody titres, and inflammatory markers in dogs affected by CanL and CKD [10].

Our present results indicate that domperidone, plus renal diet, is also able to slow the progression of renal disease in leishmaniotic dogs with CKD, using sSDMA and sCr as surrogates for the detection of renal functional impairment.

The progressive reduction in kidney function represents one of the most common and life-threatening outcomes in dogs affected by CanL, as well as a major management problem for veterinary practitioners [23]. Therefore, the early diagnosis of renal disease has assumed a key role in patient care of leishmaniotic dogs [23]. Recently, there has been increasing interest in recognizing new and early biomarkers for kidney disease and renal damage progression. Notably, sSDMA has taken a prominent position, being promising both in the prompt diagnosis of nephropathy and as a biomarker of CKD progression. The prognostic value of sSDMA and sSDMA/sCr has been previously validated [14, 24]. In particular, dogs with sSDMA of 15–19 µg/dl have a 2.5–4-fold higher risk of reduced renal function within 6 months compared to those with sSDMA ≤ 14 µg/dl [14]. Moreover, an sSDMA/sCr ratio > 10 µg/dl indicates a poor prognosis in dogs and cats with CKD [25].

In our study, after 3 months of renal diet, sSDMA decreased significantly in the dogs enrolled, confirming that dietary interventions benefit both azotemic and non-azotemic dogs at earlier stages of CKD [12, 26, 27]. Indeed, therapeutical diets represent one of the recommendations for managing CKD progression and improving the survival rate in dogs [28]. Subsequently, while maintenance on a renal diet (control group) did not prevent sSDMA from increasing again up to values similar to those at enrolment (T0), the combination of renal diet with domperidone (treatment group) allowed this renal biomarker to remain stable until the end of the trial, when it continued to be significantly lower than the enrolment value. Similarly to sSDMA, sCr remained stable in the treatment group but increased in the control group throughout the study period. These results demonstrate that treatment with domperidone, in combination with a renal diet, maintained stable renal function in the dogs enrolled, despite these having an sSDMA mean value of ≥ 15 μg/dl at enrolment. Meanwhile, it is reasonable to suppose that renal function decreased in the control group considering the progressive increase in sSDMA from T1 until the end of the study and that 80% of dogs included in group C showed an increase of more than 15% in sCr.

The main limitation of the present study is represented by the absence of proteinuria evaluation in the enrolled dogs. However, urine samples of satisfactory volume and quality are difficult to obtain from shelter dogs. Moreover, the ineffectiveness of domperidone in reducing proteinuria in leishmaniotic dogs was previously evaluated [10] and was not part of the study aims. Furthermore, although the lack of determination of proteinuria may represent a limit, the correlation of sSDMA with urinary protein/creatinine ratio (UPC) in leishmaniotic dogs has also been studied [29, 30]. More precisely, in dogs with CanL, a positive association between increased sSDMA and the presence of proteinuria and a moderate correlation between sSDMA concentration and UPC value have been described.

## Conclusion

In conclusion, domperidone plus renal diet reduces the progression of kidney disease in leishmaniotic dogs affected by CKD in comparison with renal diet alone, as demonstrated by the reduction of sSDMA and the stable sCr value evaluated during the 11-month-long field trial described herein.

## Data Availability

The datasets generated and/or analyzed during the current study are available from the corresponding author on reasonable request.

## Abbreviations

CanL: Canine leishmaniosis
CBC: Complete blood count
CKD: Chronic kidney disease
CLWG: Canine Leishmaniasis Working Group
CRP: C-reactive protein
DA_2_: Dopamine 2
GFR: Glomerular filtration rate
IFAT: Indirect fluorescent antibody test
RPF: Renal plasma flow
sCr: Serum creatinine
sSDMA: Serum symmetric dimethylarginine
sSDMA/sCR: Ratio of serum symmetric dimethylarginine to serum creatinine
UPC: Urinary protein/creatinine ratio
